# Manifestations and etiology of cutaneous findings in cases of morbid obesity

**DOI:** 10.1007/s12024-023-00721-3

**Published:** 2023-10-27

**Authors:** Roger W. Byard

**Affiliations:** Forensic Science SA and the School of Biomedicine, The University of Adelaide, Level 2, Room N237, Helen Mayo North, Frome Road, Adelaide, SA 5000 Australia

**Keywords:** Morbid obesity, Adipose tissue overload, Cutaneous manifestations, Cellulitis, Pressure ulcers, Buried penis

## Abstract

Morbid obesity is associated with a wide range of metabolic, infective, and organic disorders related to adipose tissue overload. While careful documentation of internal autopsy findings is usual, skin manifestations may be overlooked. Skin manifestations are quite diverse and include striae distensae, skin tags, plantar hyperkeratosis, acanthosis nigricans, the sequelae of hyperandrogenism, lymphedema, panniculus morbidus, chronic venous insufficiency, stasis dermatitis, leg ulceration, intertrigo, cellulitis, pressure ulcers and ‘buried penis’. Obesity has also been associated with hidradenitis suppurativa, psoriasis, atopic dermatitis, melanoma, systemic lupus erythematosus, lichen planus and acne vulgaris. Evaluating these findings at the time of autopsy may give a more complete assessment of a particular case and may also identify conditions that may have contributed to, or caused, death.

## Introduction

Obesity is an increasing problem globally with estimates that adults in 199 countries over the period 1980 to 2008 had increases in body mass index of 0.4 kg/m^2^/decade (BMI = weight in kilograms divided by the height in meters squared). Leading countries were the USA, Australia and New Zealand [1, 2].

Although there have been efforts recently to downplay the significance of obesity, suggesting that it is more a lifestyle choice rather than a condition, it is linked to a wide range of negative medical consequences with the current trends around the world being referred to as an ‘obesity epidemic’ [3]. Adverse associations include not only cardiovascular diseases but respiratory and endocrine disorders all associated with metabolic derangements arising from adipose tissue overload [4, 5]. There is also an increased risk for sepsis and certain malignancies, including those of the breast, uterus, cervix, ovary, liver, gallbladder, esophagus, stomach, colon, rectum, pancreas, kidney, and thyroid, with an increased incidence of leukemia, non-Hodgkin lymphoma, and multiple myeloma [6–8]. Thus, at the time of autopsy, there are a plethora of potential underlying natural conditions to be considered and evaluated [4].

In addition, obesity is associated with a variety of serious injuries and unnatural deaths ranging from increased risks for leg amputations in motor cycle riders to lethal positional asphyxia [9, 10]. The post-mortem handling of morbidly obese bodies can also be difficult due to problems in lifting, handling and storing, and then in subsequently adequately examining large, prematurely-putrefied bodies [11].

As well as organic illnesses caused by obesity, at the time of external examination, there may be a number of characteristic skin changes and lesions which are present often associated with insulin resistance, hyperandrogenism and the mechanical and microenvironmental effects of skin folds [4]. As these are not always considered in the forensic evaluation of cases, the following review was undertaken to document and analyze typical cutaneous markers of morbid obesity and to explore their possible etiology. It is recognized that there are also a wide range of conditions where obesity is a result of an underlying disorder including such entities as Cushing disease, Prader Willi syndrome and adiposa dolorosa (previously known as Dercum disease). However, these have not been discussed as this overview is focussing instead on cutaneous manifestations that arise from simple adipose tissue overload (ATO).

## Striae distensae

**Fig. 1:**
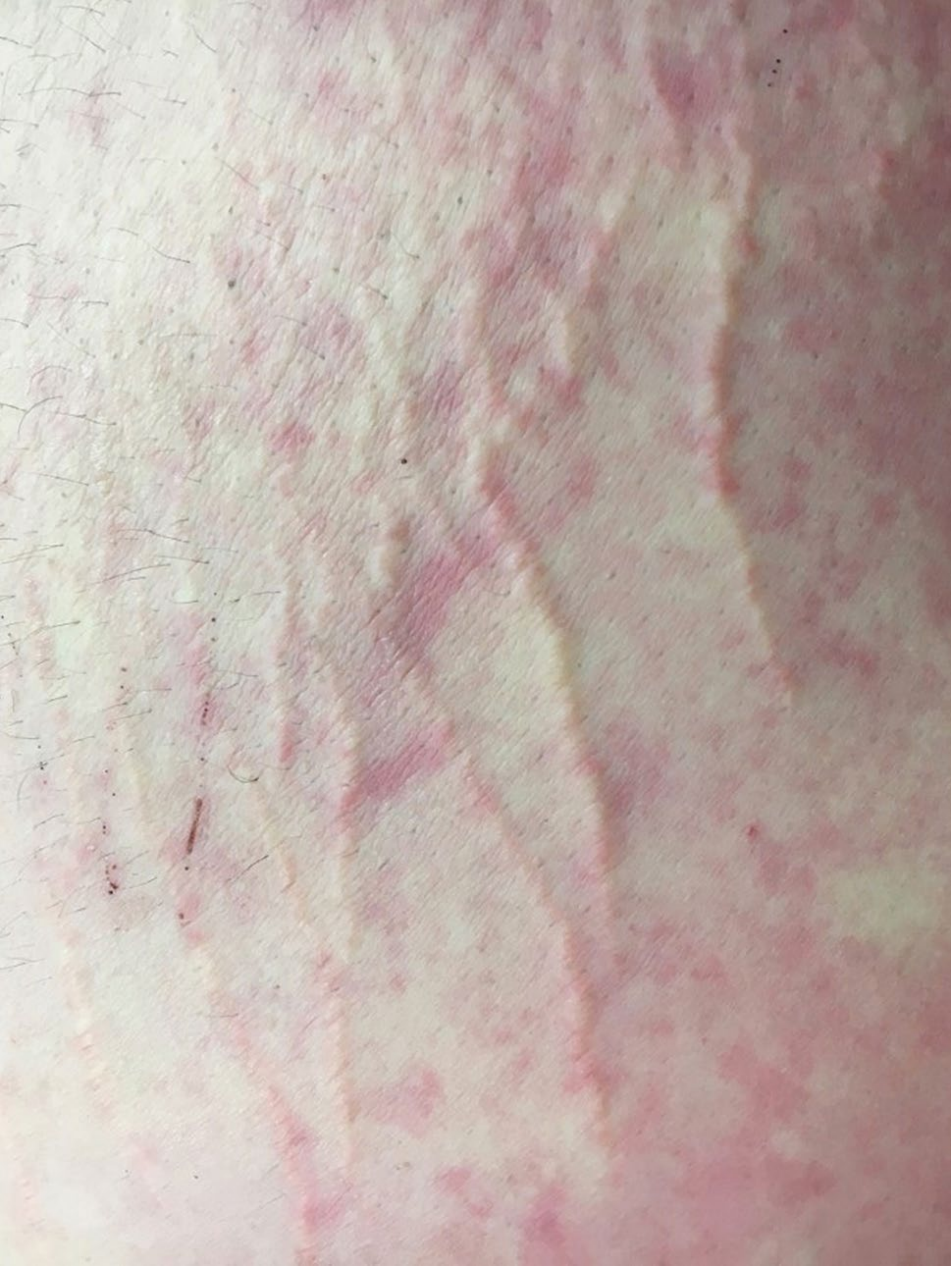
Elevated striae in the lower abdomen in a case of morbid obesity

One of the first changes to the skin that is apparent is the presence of widespread atrophic linear marks, striae or ‘stretch marks’ which have been found in 43% of obese individuals [12]. They were first described histologically in 1889 and occur predominantly in the skin of the abdomen, buttocks, thighs and arms. The acute stage, termed striae rubra, is characterized by markings that are red to violaceous in colour, raised and lying perpendicular to skin tension lines (Fig. 1). Over time, the chronic state of striae alba develops where the lesions are atrophied, white and depressed [13].

The etiology of striae is not completely understood, with suggestions that they may be caused by elevated steroid levels (being seen in Cushing syndrome, pregnancy and after steroid therapy), possibly in combination with rapid stretching of the skin [14]; the latter supported by their positioning relative to lines of maximum skin tension. There may be a genetic predisposition. It is likely that there is stretching and thinning of underlying connective tissues with increasing underlying adipose tissue accumulation which causes elastin and collagen fibres to rupture with scarring and new collagen deposition [15, 16].

Histological evaluation of early lesions shows dermal edema with perivascular lymphocytic cuffing, increased melanogenesis and vascular ectasia of the papillary dermis. There is a reduction in elastin with alteration of collagen in the reticular dermis. In contrast striae alba show atrophy with decreased vascularity, loss of rete ridges, hair follicles and other appendages, and an accumulation of glycosaminoglycans. Striae are clearly separated from adjacent normal skin by a layer of eosinophilic collagen bundles [13, 16, 17].

### Skin tags

**Fig. 2:**
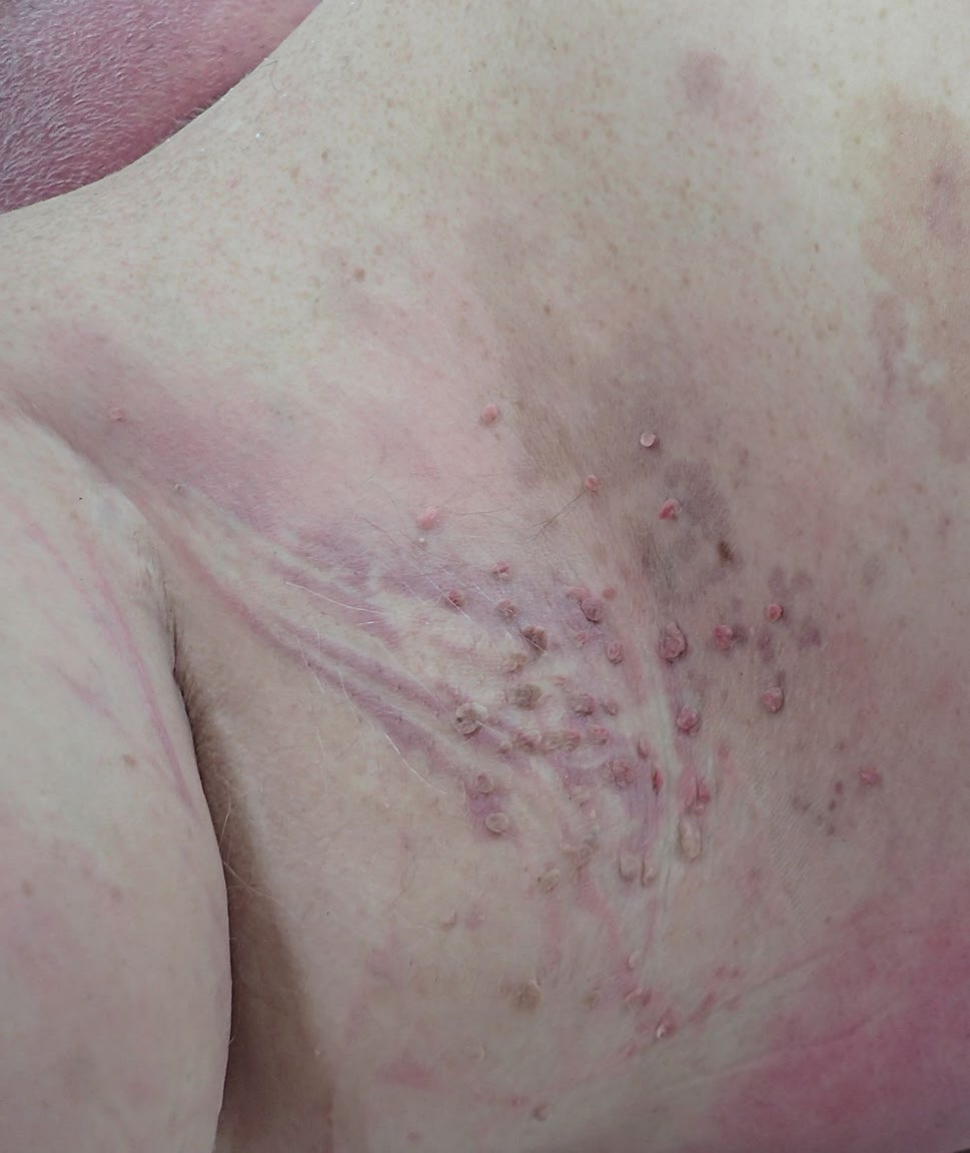
A cluster of axillary skin tags in a case of morbid obesity

Skin tags are another finding that is common in obese individuals, occurring in up to 44% of cases [12]. They consist of small, fleshy, pedunculated lesions, most often in the axillae (Fig. 2), neck or eyelids, often associated with acanthosis nigricans. They are more common in women and increase in numbers as the BMI increases [18].

The cause of increased numbers of skin tags in obese individuals is thought to be related to complex metabolic derangements associated with hyperinsulinemia and increased leptin levels [19, 20]. Increasing body fat may decrease the sensitivity to insulin receptor substrate resulting in increased pancreatic production of insulin and resultant higher levels of insulin growth factor, which in turn stimulates skin and fibroblast proliferation [21].

### Plantar hyperkeratosis

This refers to marked thickening of the stratum corneum of the skin of the heel, arch and over the metatarsophalangeal joints. It has been found to be one of the most common skin manifestations of obesity, being found in nearly 50% of obese people, the incidence going up with increasing BMI [22]. It is related to increased pressure and friction on the sole of the foot, in particular the metatarsal heads, from the effects of excessive weight [13, 23].

### Acanthosis nigricans

Acanthosis nigricans refers to dark areas of hyperpigmented skin with irregular borders in body folds that are described variably as velvety, papillomatous or leathery. Most often found in the axillae, it may also occur on the back of the neck, in the scalp, the groin, or over the elbows and knees [24]. It has a well-established association with internal malignancies such as gastric cancer and endocrine disorders such as diabetes mellitus, and may on occasion be familial [25–28]. In addition, it has been documented in obesity [29] occurring in 33% of obese individuals who are 120–170% above their ideal body weight, increasing to 100% in those greater than 250% [18].

Although the precise etiology is unclear, it appears that these areas of hyperplastic skin result from the effects of hyperinsulinemia stimulating insulin-like growth factor causing proliferation of dermal fibroblasts and keratinocytes [30]. Histologically, there is papillomatosis and hyperkeratosis with minimal hyperpigmentation and usually no inflammation unless there is a superimposed intertriginous infection.

### Features of hyperandrogenism

Obesity and its associated metabolic consequences can lead to an increase in the production of androgens from the ovaries due to hyperinsulinemia. In addition, adipose tissue produces testosterone, all of which may lead to elevation of serum testosterone in obese females with resultant hirsutism, acne and male-pattern baldness [31, 32].

### Lymphedema

**Fig. 3:**
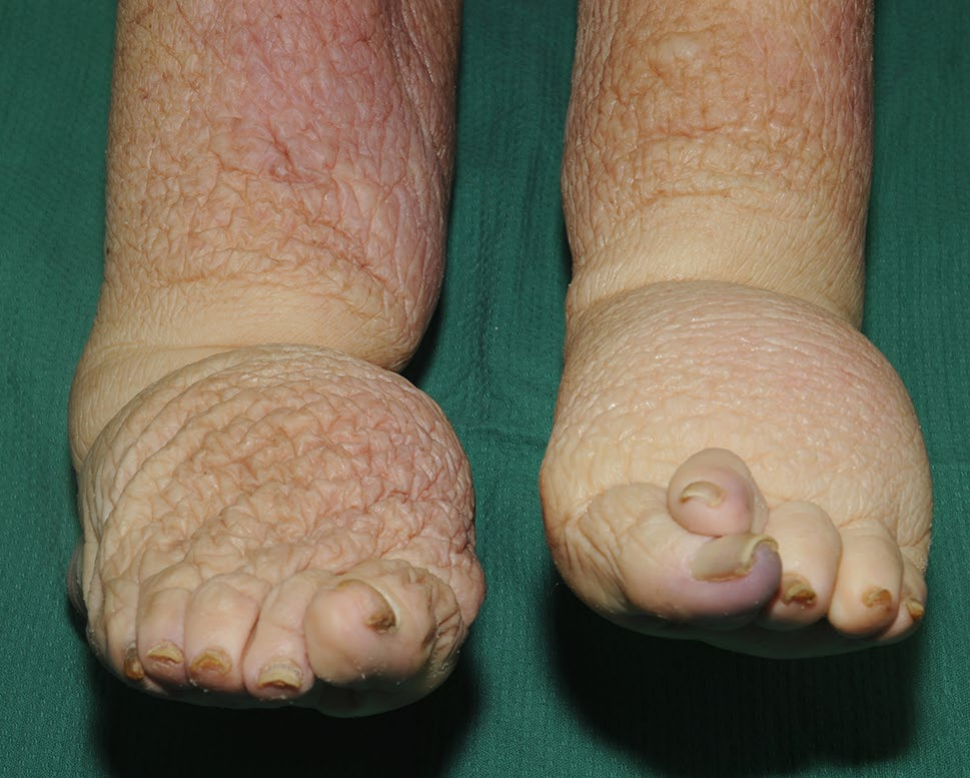
Chronic lymphedema of the lower legs and feet in a case of morbid obesity

Lymphedema is the term used for swelling of the tissues due to accumulation of fluid that is normally drained by the lymphatics. It may be primary or secondary, occurring in the legs (Fig. 3) and arms most commonly, but may also be found in the genitalia and abdominal wall. Secondary lymphedema is a very common complication of obesity occurring in over 75% of morbidly obese individuals. Obesity-induced lymphedema (OIL) increases with elevated BMI and is almost always present in individuals with BMIs over 60 [33–35].

The etiology of OIL has been debated, as recovery often does not occur after weight has been lost, suggesting that permanent damage to the lymphatics has occurred [36]. It appears likely that massive adipose tissue accumulation compresses lymph channels obstructing fluid return, which in turn further exacerbates external pressure on the walls of lymph channels. Pooled, protein-rich fluid in the soft tissues reduces tissue oxygenation and acts as an excellent growth media for bacterial colonization and proliferation leading to an inflammatory response with scarring of lymphatic vessels with further obstruction exacerbating lymphedema [13]. Soft tissues also scar with fibrosis and thickening of the overlying skin increasing the vulnerability to injury.

**Fig. 4:**
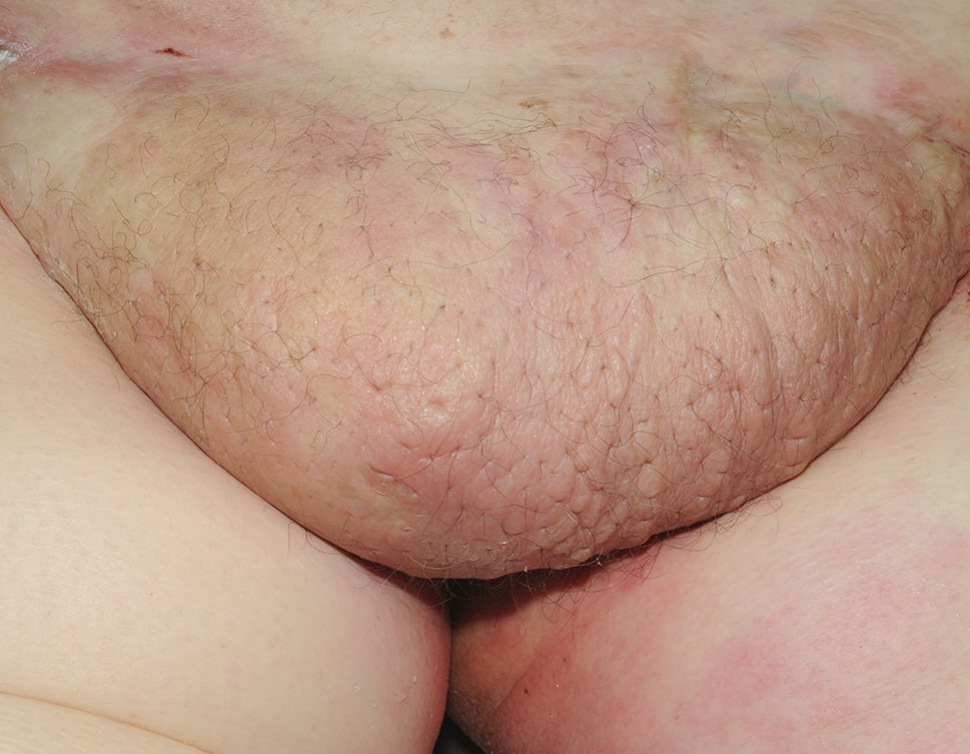
Lymphatic obstruction of the fatty apron of a morbidly obese individual, so-called panniculus morbidus, with typical peau d’orange changes

Lymphatic obstruction in the fatty apron of individuals with morbid obesity can also result in significant lymphedema, or so called panniculus morbidus. In these individuals, yet another vicious cycle may then be set up with the increasing weight of the sodden anterior abdominal wall tissues causing them to be drawn downwards by gravity. This then leads to more fluid accumulation from further impaired lymphatic and venous drainage [37]. The skin often has a classic ‘peau d’orange’ appearance due to the swollen tissues being focally tethered by skin appendages giving it a dimpled appearance (Fig. 4). The size of the abdominal pannus can be ranked from Grade 1—covering the pubic hairline, but not the entire mons pubis, through to Grade 5—extending to the knee and below [38].

### Chronic venous insufficiency

**Fig. 5:**
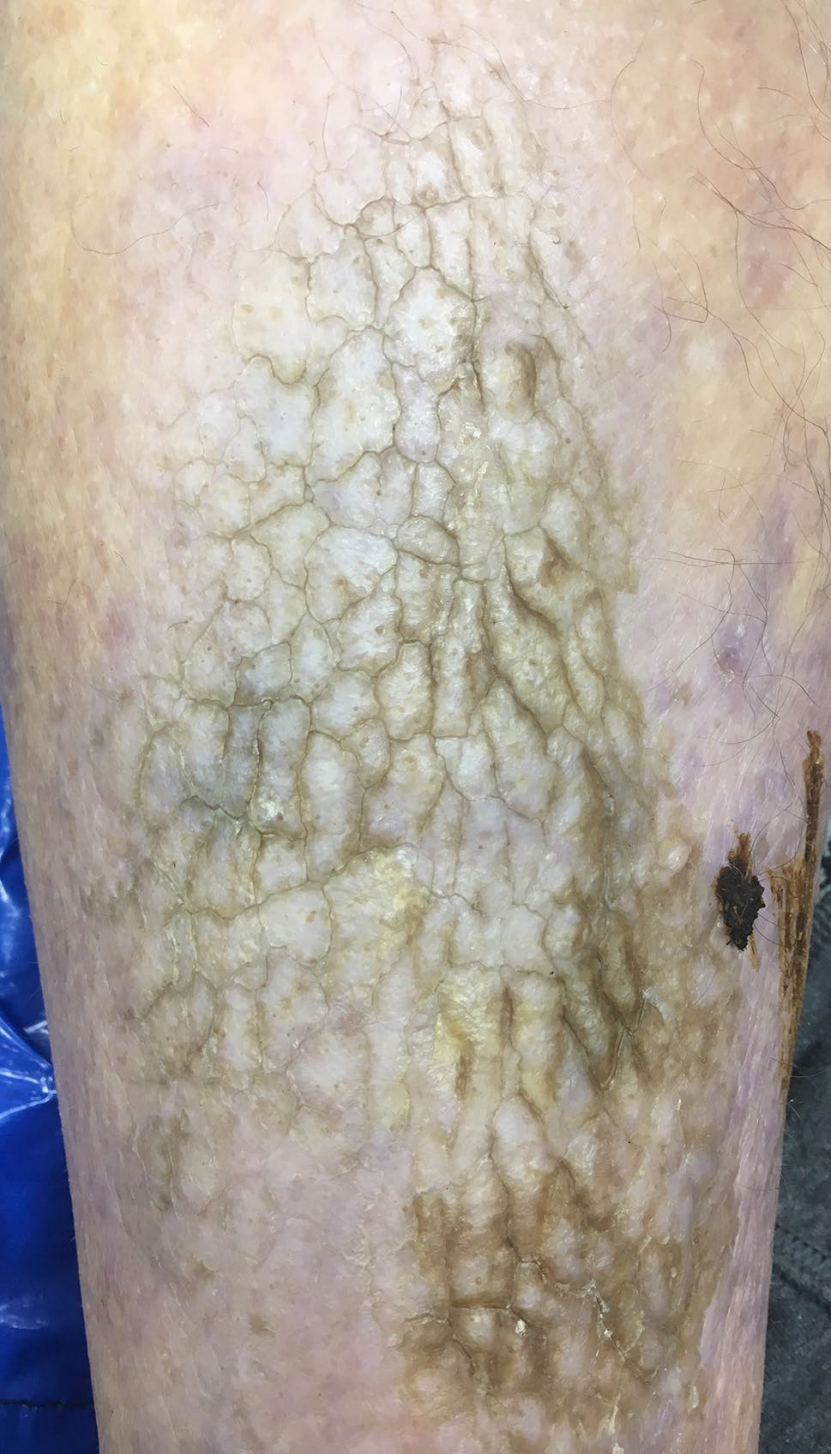
Violaceus discoloration with skin crusting and cracking in a case of chronic venous insufficiency in a case of morbid obesity

Chronic venous insufficiency is a common finding in cases of obesity resulting in extravasation of fluid and blood cells into the interstitium of the lower legs. Problems with lymphatic drainage noted above reduce the amount of leaked fluid that can be removed, resulting in swelling and pitting edema that can be readily identified at autopsy. Stasis dermatitis may develop with brown/violaceous discoloration of the skin that may be dry and crusted with cracking [13] (Fig. 5).

Venous insufficiency occurs in obesity due to increased intra-abdominal pressure resulting in an obstructed venous return, which in turn leads to valvular insufficiency in the long veins of the leg further worsening the situation. An additional compounding factor is the presence of a low grade inflammatory response in obesity associated with increased circulating levels of plasminogen activator inhibitor-1, tumor necrosis factor-alpha, interleukin, leptin and C-reactive protein [4]. This causes damage to the veins exacerbating insufficiency and predisposing to thromboembolism [39]. Varicose ulceration may develop [4].

### Intertrigo/mechanical friction

Intertrigo refers to inflammation of the skin in folds where there has been friction between the opposing surfaces with increased sweating and the accumulation of moisture. The areas may be quite erythematous and macerated with weeping and crust formation [40]. There is a direct association with obesity as the increased surface area of body folds creates more areas for friction to occur; obese patients sweat more into body folds and then retain moisture, and the skin has higher alkalinity with a BMI > 25 that predisposes to superinfection by candida species [13]. Increased sweating is also predisposed to by overheating due to the insulating effects of increased subcutaneous fat.

**Fig. 6:**
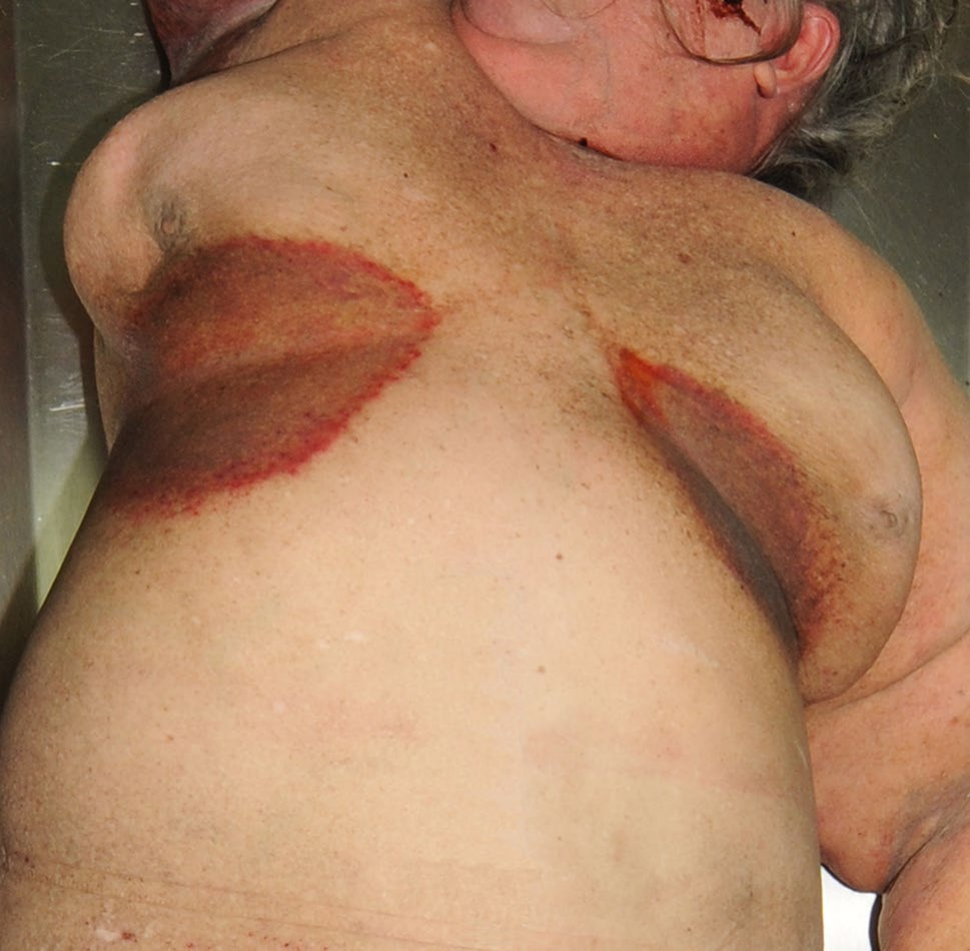
Bilateral submammary reddened and moist areas of intertrigo in a case of morbid obesity

The warm, dark and humid environments of the anogenital, umbilical, axillary and sub-mammary areas (Fig. 6), added to the layers of compressed, desquamated skin, provide a rich environment for bacterial and fungal colonization. It may also occur between the toes. A commonly found organism is *Candida albicans* which flourishes when there may be difficulty in adequately cleaning extensive and folded skin surfaces [4]. Insulin resistance leading to diabetes mellitus may further enhance bacterial and fungal proliferation [41].

In obese females, the perineum may be a particularly vulnerable area as increased amounts of intra-abdominal fat may compress the bladder causing urinary incontinence resulting in the area being permanently moist [14]. A much more serious complication is Fournier gangrene, a rapidly progressive necrotizing fasciitis of the perineum and perianal region. The most common causative organism is *Escherichia coli* although *Bacteroides*, *Streptococci* and *Clostridia *sp. may also be involved [42]. Risk factors include obesity and diabetes mellitus with an associated mortality rate of 45% [43, 44].

### Infections

Obesity is associated with increased rates of infection, even after controlling for diabetes mellitus, most likely due to interactions of pro- and anti-inflammatory factors with immunosuppressive effects. As noted above, lymphedema predisposes to bacterial infections with morbid obesity being associated with an over two and half times increase in the occurrence of cellulitis [45]. Similarly, humid microenvironments within intertriginous folds encourage micro-organism growth, particularly in areas that may be difficult to clean.

### Pressure ulcers

**Fig. 7:**
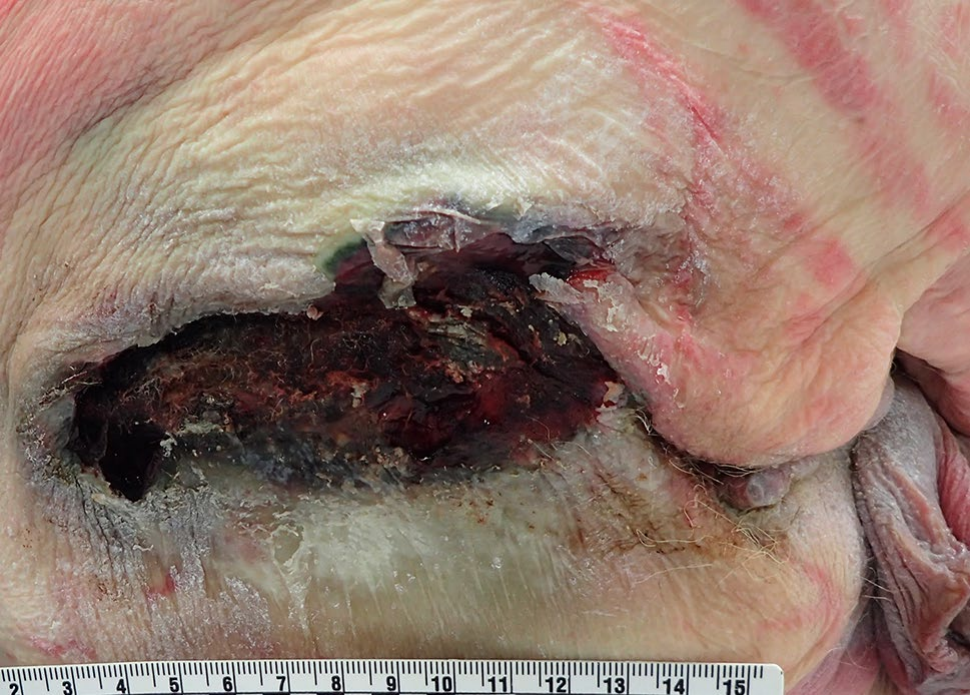
A typical area of decubitus/pressure ulceration over the sacrum

**Fig. 8:**
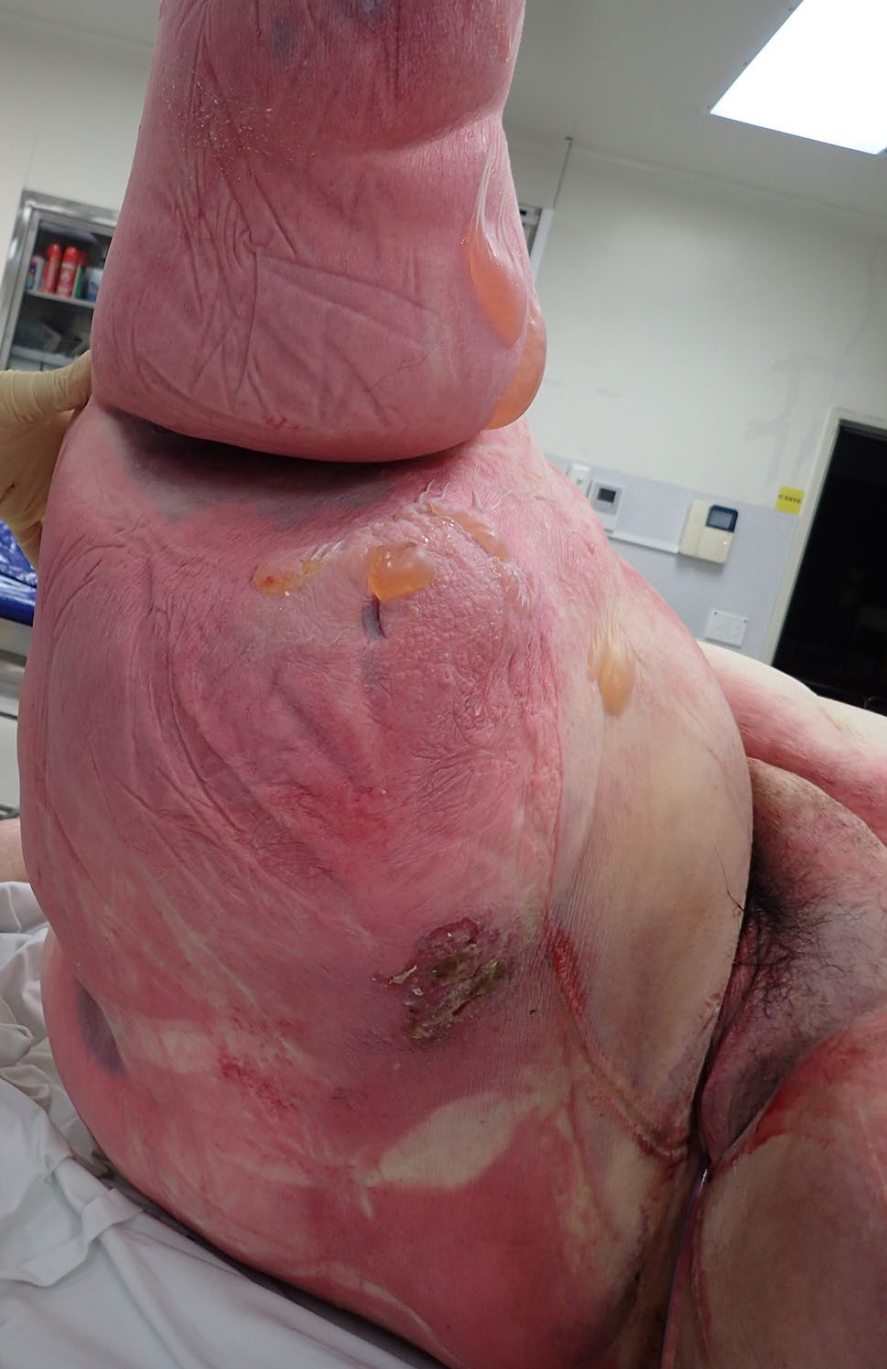
An unusual site with an early pressure sore under the thigh in a morbidly obese woman, also showing changes of early decomposition with skin blistering

Pressure/decubitus ulcers or bedsores are areas of necrosis of the skin and underlying tissues caused by prolonged pressure. They occur in debilitated individuals who are unable to move regularly and are usually found in skin that covers bony areas such as the sacrum (Fig. 7), heels, ankles and hips [46]. Morbid obesity is associated with the development of pressure sores due to difficulties in moving in bed and also to significant underlying comorbidities. Unusual sites for pressure ulcers that should be checked for at autopsy in the obese include within skin folds due to capillary compression with tissue necrosis, and also under the thighs (Fig. 8), or over the hips due to prolonged compression from bed rails or the sides of wheelchairs [14, 47].

### Buried penis

**Fig. 9:**
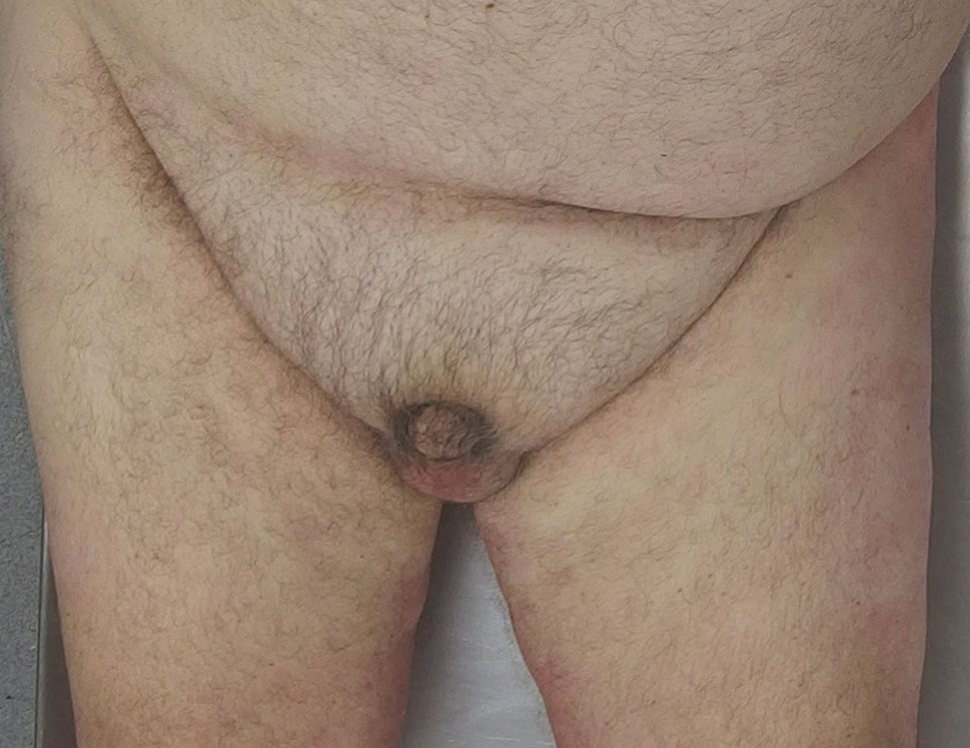
A ‘disappearing penis’ being enveloped by pudendal fat in a case of morbid obesity

Buried penis refers to the situation where the penile shaft has become enveloped by encroaching suprapubic adipose tissue in morbid obesity [48] (Fig. 9). It differs from a micropenis as the penis is usually of normal length and has been found in 5% of cases of morbid obesity [49]. The forensic significance is that it may be associated with complications such as bacterial and fungal infections due to chronic moistening of the area and issues with hygiene. This may lead to scarring with urethral stricture formation and urinary tract obstruction [50]. There may also be premalignant changes with 7% of individuals having established invasive penile cancer [51, 52]. Thus, the area should be carefully assessed at autopsy for any of these complications.

### Other associations

An elevated BMI has been found to be an independent risk factor for hidradenitis suppurativa, the severity of psoriasis and its response to systemic therapy, atopic dermatitis and melanoma. Obesity also has an association with systemic lupus erythematosus, lichen planus and acne vulgaris [53].

## Conclusions

Morbid obesity is accompanied by a wide range of skin lesions resulting from the underlying metabolic and infective complications of adipose tissue overload. Documentation of these findings at the time of autopsy provides a more complete assessment of the manifestations of obesity and may also provide insight into conditions that may have contributed to death.

## Key points

1. Morbid obesity is linked to a wide range of metabolic, infective and organic disorders associated with adipose tissue overload.
2. Skin manifestations have quite diverse manifestations and etiology.
3. Evaluating these findings at the time of autopsy may give a more complete assessment of a particular case.
4. Certain of these conditions may contribute to death.
